# Ants farm subterranean aphids mostly in single clone groups - an example of prudent husbandry for carbohydrates and proteins?

**DOI:** 10.1186/1471-2148-12-106

**Published:** 2012-07-02

**Authors:** Aniek BF Ivens, Daniel JC Kronauer, Ido Pen, Franz J Weissing, Jacobus J Boomsma

**Affiliations:** 1 Theoretical Biology, Centre for Ecological and Evolutionary Studies, University of Groningen, Nijenborgh 7, Groningen 9747 AG, The Netherlands; 2 Centre for Social Evolution, Department of Biology, University of Copenhagen, Universitetsparken 15, Copenhagen DK-2100, Denmark; 3 Laboratory of Insect Social Evolution, The Rockefeller University, 1230 York Avenue, New York, NY, 10065, USA

**Keywords:** Cultivation mutualism, Host-symbiont conflict, Symbiont competition, Monoculture, Clonal mixing

## Abstract

**Background:**

Mutualistic interactions are wide-spread but the mechanisms underlying their evolutionary stability and ecological dynamics remain poorly understood. Cultivation mutualisms in which hosts consume symbionts occur in phylogenetically diverse groups, but often have symbiont monocultures for each host. This is consistent with the prediction that symbionts should avoid coexistence with other strains so that host services continue to benefit relatives, but it is less clear whether hosts should always favor monocultures and what mechanisms they might have to manipulate symbiont diversity. Few mutualisms have been studied in sufficient genetic detail to address these issues, so we decided to characterize symbiont diversity in the complex mutualism between multiple root aphid species and *Lasius flavus* ants*.* After showing elsewhere that three of these aphid species have low dispersal and mostly if not exclusively asexual reproduction, we here investigate aphid diversity within and between ant nest mounds.

**Results:**

The three focal species (*Geoica utricularia*, *Forda marginata* and *Tetraneura ulmi*) had considerable clonal diversity at the population level. Yet more than half of the ant mounds contained just a single aphid species, a significantly higher percentage than expected from a random distribution. Over 60% of these single-species mounds had a single aphid clone, and clones tended to persist across subsequent years. Whenever multiple species/clones co-occurred in the same mound, they were spatially separated with more than 95% of the aphid chambers containing individuals of a single clone.

**Conclusions:**

*L. flavus* “husbandry” is characterized by low aphid “livestock” diversity per colony, especially at the nest-chamber level, but it lacks the exclusive monocultures known from other cultivation mutualisms. The ants appear to eat most of the early instar aphids, so that adult aphids are unlikely to face limited phloem resources and scramble competition with other aphids. We suggest that such culling of carbohydrate-providing symbionts for protein ingestion may maintain maximal host yield per aphid while also benefitting the domesticated aphids as long as their clone-mates reproduce successfully. The cost-benefit logic of this type of polyculture husbandry has striking analogies with human farming practices based on slaughtering young animals for meat to maximize milk-production by a carefully regulated adult livestock population.

## Background

Mutualistic symbioses are widespread and of crucial importance in many ecosystems [1]. Although evolutionary theory to explain the stability of mutualistic interactions has progressed considerably (see [2] for a review), consensus on the general underlying mechanisms that keep these interactions stable and cooperative has not been achieved [3-7]. While further theoretical work might alleviate this problem, these difficulties also illustrate that mutualistic interactions are highly variable in their ecological contexts [8-10] and degrees of commitment [11-13], and that very few of them have been studied in considerable depth (reviewed in [2]). Two aspects are thought to have important implications for the interaction stability of host-symbiont mutualisms:

1. The level of sexual reproduction and the degree of independent dispersal of the symbionts, and

2. Genetic diversity among symbionts of a single host [3]. In a previous study we investigated the first aspect in the hitherto poorly studied mutualism of *Lasius flavus* ants farming root-aphids [14]. The present study focuses on the second aspect.

In cultivation (farming) mutualisms, the host partner promotes the growth of a symbiont that it consumes, either individually or as somatic modules [15]. While scenarios of ‘enslavement domestication’ have been suggested for the early evolution of such mutualisms [16,17], it remains difficult to understand how symbionts would be actively selected to make the transition from free-living to being domesticated. The latter state would imply becoming reproductively isolated from free-living relatives which would require consistent direct benefits to be sustainable. Domestication often also implies losing options for horizontal transmission, having many offspring consumed by the host, and potentially being mixed with other symbiont lineages, consequences that could all discourage life as a symbiont. Domestication mutualisms would thus seem most likely to evolve if symbiont services ultimately benefit the reproduction of close symbiont relatives and if the productivity of domesticated reproduction consistently exceeds the fitness that can be obtained from a free-living life-style. When symbionts are already clonal before domestication, one would therefore expect symbioses to elaborate this form of propagation when making symbionts commit irreversibly to a dependent life-style, which requires new host-serving adaptations that impede survival and reproduction without the host. The ‘trophobiotic organs’ evolved in the aphids of our present study [18,19] are examples of such adaptations.

While symbiont interests in being cultivated would be expected to benefit from monopolizing host attention to a group of close relatives, hosts should not necessarily favor the same tendencies towards rearing monocultures, as a more variable community of symbionts might offer a broader spectrum of services or be less vulnerable to parasites (e.g. [20]). As outlined by in earlier studies [21,22], hosts would be selected to enforce monocultures only if scramble competition between multiple symbiont strains would decrease the overall productivity of the symbiotic interaction, i.e. if different symbiont strains would compete for the same limited resource provided by the host. Similar selection pressure towards monoculture farming would apply if coexistence of multiple strains within the same host would allow free-riding by underperforming strains, leading to a direct reduction in overall productivity (e.g. [10,23]).

Incentives for competition or cheating would destabilize mutualistic interactions between symbionts and hosts, unless specific mechanisms of symbiont screening upon admission [24] or symbiont rewarding/sanctioning in proportion to performance [5,10] can evolve. The relative importance of these mechanisms is controversial, but available data suggest that monocultures are commonly found in the cultivation mutualisms that have been studied, from the gardens of algae-growing damselfish [25] to those of fungus-growing termites and ants [11,26-28]. In fungus-farming leaf-cutting ants, monocultures appear to be enforced by a combination of incompatibility between genetically different symbiont strains and active symbiont policing by the hosts [11,28,29], whereas a simple mechanism of positive frequency-dependent propagation within established colonies appears sufficient to enforce life-time commitment between a termite host colony and a single symbiont clone [27]. However, more studies are needed to establish the generality of this principle, particularly for cultivation mutualisms where hosts are able to segregate symbionts in space or time to avoid competition [9], so that the benefits of polyculture might surpass the costs.

In the present study we focus on a farming symbiosis that has been known for decades but has rarely been studied: the root aphid husbandry for sugar (honeydew, “milk”) and nitrogen (“meat”) of the Yellow meadow ant *Lasius flavus*, which is likely to be essential for ant colony growth and reproduction, and involves an entire array of root aphid species [18,30-35]. These root aphid species have a number of distinct traits that improve performance as ant symbionts but are never found in free-living aphids, such as the ‘trophobiotic organ’ to hold honeydew for the ants [19]. The most common species have further lost most if not all sexual reproduction in Northwest Europe, but have maintained low frequencies of winged morphs that may disperse between colonies [14]. In the present study we use a newly developed set of DNA microsatellite markers [36] to assess aphid species number and clonal diversity at the level of single ant nest mounds.

**Figure 1:**
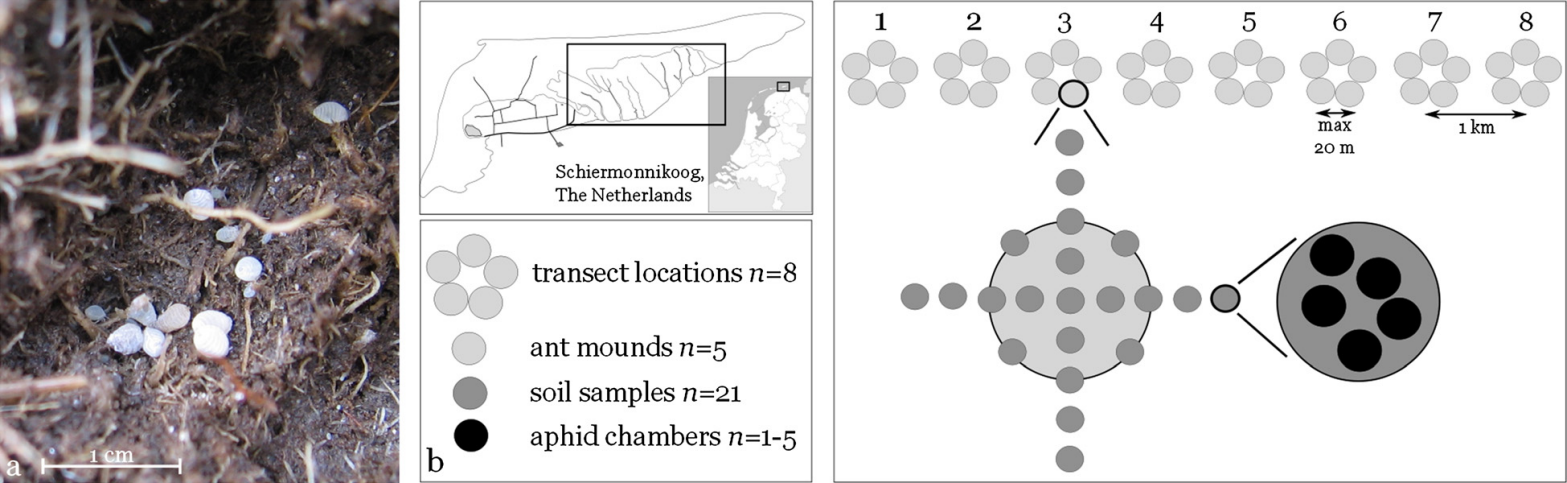
**The sampling scheme for root aphids in nest mounds of the ant *Lasius flavus. *a.** A representative large aphid chamber with many, mostly adult, *Geoica utricularia*, **b.** Aphids were sampled from ant mounds on the island of Schiermonnikoog (The Netherlands) along a transect on the salt-marsh (framed area on map, corresponding to the area shown in Figure 2). Sampling was done in a nested design with four levels. At every transect location (level 1, location 1–8), we sampled 5 ant mounds (level 2), by taking 21 soil samples (level 3), located in, on the edge of, or just outside an ant mound. The collected aphids within each sample were kept separate per aphid chamber (level 4). (Photo: A.B.F. Ivens, maps courtesy of D. Visser).

The objectives of our study were to use hierarchical sampling (Figure 1) and DNA microsatellite analysis to: 1. Estimate species- and clone diversity for three focal species of root aphids (*Geoica utricularia, Tetraneura ulmi, Forda marginata*) within *L. flavus* nests, soil samples within nests, and single aphid chambers (Figure 1a) within these soil samples, 2. Evaluate whether the observed distributions are consistent with the expectation that symbiont diversity within nests is low, 3. Analyze the extent to which diversity patterns change across sampling levels and years, and 4. Infer which potential mechanisms can lead to the observed diversity patterns.

## Results

### Aphid diversity and abundance

**Figure 2:**
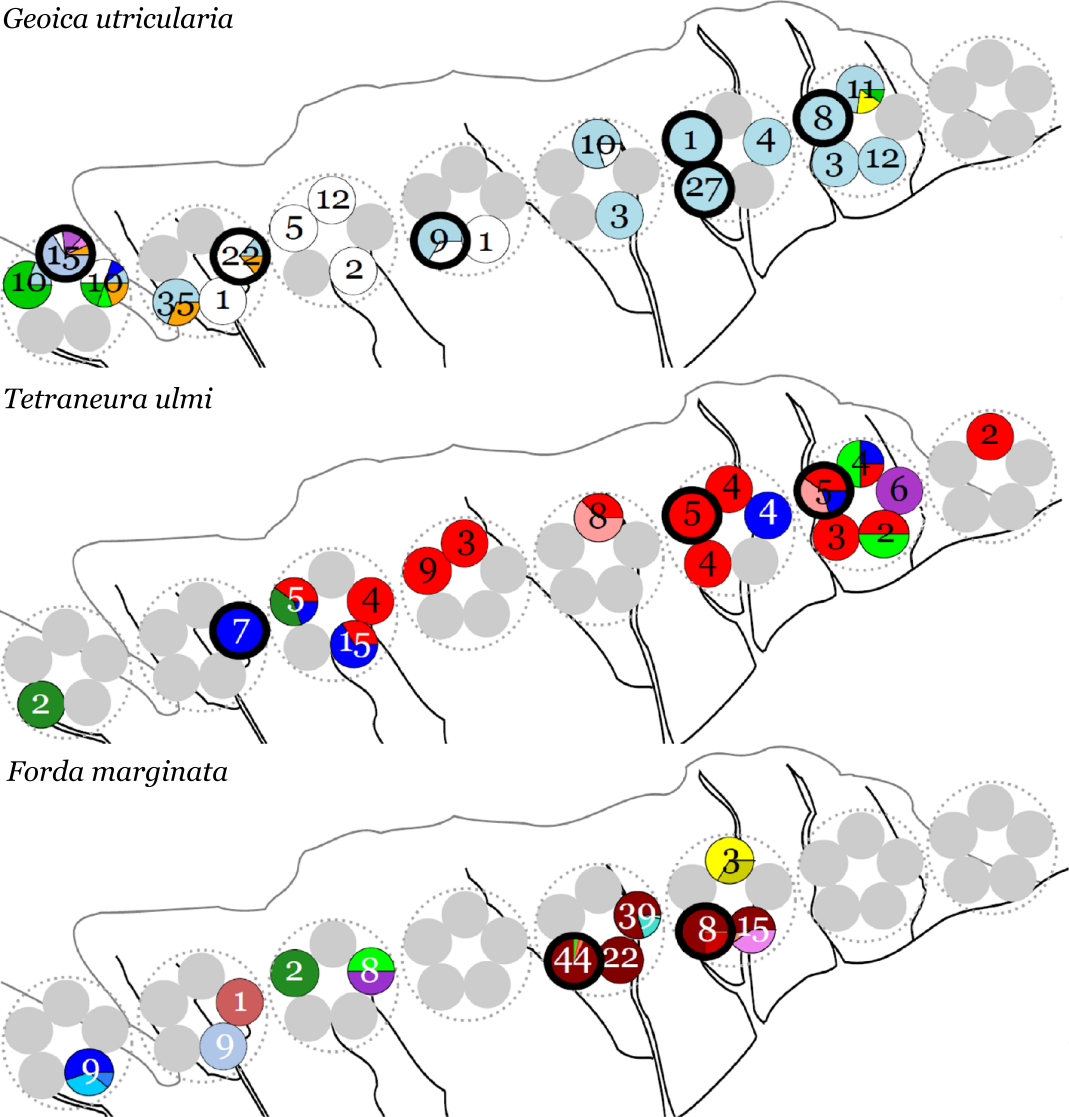
**Distribution of aphid clonal lineages per ant mound.** Data are shown for three root aphid species *Geoica utricularia* (top), *Tetraneura ulmi* (middle) and *Forda marginata* (bottom) in 2008. Large dotted circles refer to sampling locations (1–8 from left to right), whereas small filled circles refer to sampled ant mounds, with the number of aphids found in the mound indicated by numbers within circles. Colors indicate the proportion of aphids belonging to particular clonal multilocus genotypes (MLGs), whereas multilocus lineages (MLLs) that combine closely related MLGs are identifiable by their similar color shades. Mounds with bold black margins were resampled in 2009 and 2010.

As shown in Figure 2, considerable aphid diversity existed along the sampled 7 km transect, but the distribution of this diversity across ant mounds deviated significantly from random. At all sampling levels (ant mound, soil sample and chamber) monocultures containing only a single species occurred much more often than expected from a random distribution (Figure 2, Table 1), with 52% of the sampled mounds and 99% of the aphid chambers containing only a single species. Also genetic diversity within species was always non-randomly distributed over the mounds, as there were more mounds that contained a single multilocus lineage (MLL) than expected based on the distribution of MLLs over transect locations (Figure 2, Table 1). The same was true for the distribution of multilocus genotypes (MLGs) over mounds, with *G. utricularia* MLGs occurring significantly more often in monocultures than expected. In the other two species the frequency of MLG-monocultures across mounds was not significantly different from random expectation (Figure 2, Table 1).

**Table 1:** Results of the monoculture analyses

**Level**	**Ant mounds**			**Soil samples**			**Chambers**		
	***n***	**% monocultures**	**P**	***n***	**% monocultures**	**P**	***n***	**% monocultures**	**P**
**Between species**	31	52	**0.001**	145	94	**0.001**	239	99	**0.001**
**Within species MLL**									
*Geoica utricularia*	20	60	**0.028**	75	88	**0.005**	125	95	0.949
*Tetraneura ulmi*	18	72	**0.043**	39	90	0.068	50	96	1.000
*Forda marginata*	11	64	**0.015**	40	88	0.094	66	95	0.663
**Between species MLG**									
*Geoica utricularia*	20	60	**0.027**	75	88	**0.002**	125	95	0.962
*Tetraneura ulmi*	18	67	0.082	39	87	0.056	50	94	1.000
*Forda marginata*	11	36	0.099	40	73	0.707	66	88	1.000

For each organization level (between species and between MLLs and MLGs within species) the probability (P) that the observed number of monocultures at a given sampling level (ant mounds, soil samples and aphid chambers) could have resulted from a random distribution of aphids was estimated using a bootstrap approach with 1000 iterations. P values below 0.05 (bold Figures) indicate deviations from a random distribution.

**Figure 3:**
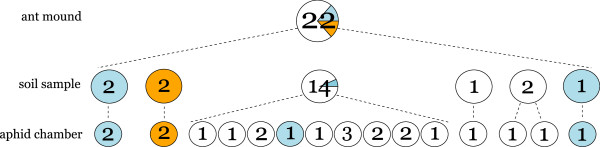
**Spatial distribution of *Geoica utricularia *MLGs in a single, representative nest mound of *Lasius flavus *.** The top pie chart gives the observed MLG distribution in the entire mound, the mid-level pie charts give the MLG distribution over soil samples, and the lower pie charts give the MLG distributions over nest chambers. Numbers indicate sample sizes per unit.

**Figure 4:**
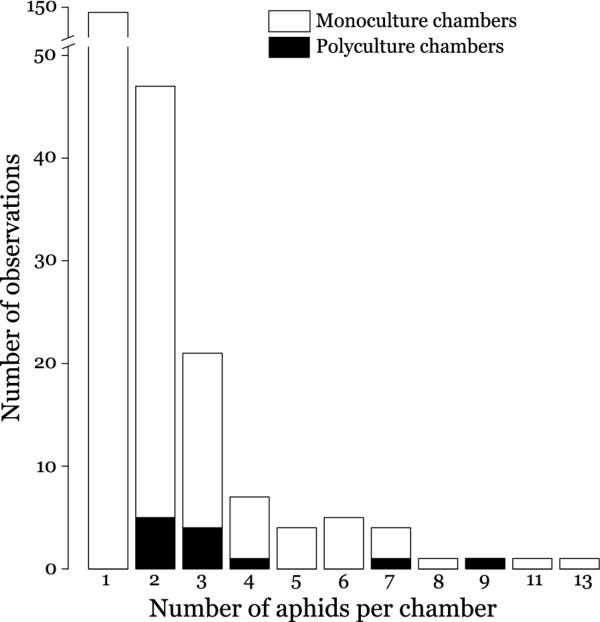
**Distribution of aphids over aphid chambers in *Lasius flavus *mounds in 2008.** Aphid numbers per chamber with genetic monocultures, i.e. aphids of the same MLL (white bars) and polycultures, i.e. chambers with aphids of multiple MLLs (black bars) (n = 239). Chambers with only a single aphid are monocultures by default.

At lower sampling levels within mounds (soil samples, chambers) high percentages of monocultures were also found, both between and within species (Table 1). However, these monoculture percentages did mostly not significantly deviate from randomness, because low aphid diversity at the species, MLL or MLG level across mounds or soil samples will automatically lead to low aphid diversity at the next level below. Figure 3 illustrates this for the spatial distribution of *G. utricularia* MLGs in one of the nests of Figure 2, showing that most MLGs occurred spatially separated already at the soil sample level, so that aphid chambers could only contain monocultures (Table 1, Figure 3). Figure 4 shows the distribution of aphid numbers per chamber, with most chambers containing only one aphid, but some chambers having as many as 13 aphids (means per chamber ± SE *G. utricularia* 1.61 ± 0.13, *T. ulmi* 1.84 ± 0.18, *F. marginata* 2.39 ± 0.28). Even aphid chambers with rather many aphids often contained monocultures in terms of MLLs (Figure 4), and chambers that did contain polycultures never had more than 2 MLLs.

### Annual turnover of aphid clonal lineages

**Figure 5:**
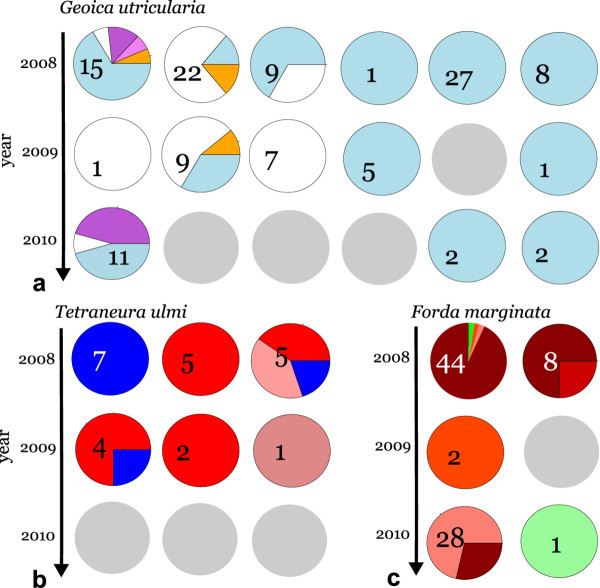
**Temporal variation of aphid clones in *Lasius flavus *mounds over three years (2008, 2009, 2010).***Geoica utricularia* (**a**), *Tetraneura ulmi* (**b**), and *Forda marginata* (**c**). Colors indicate MLGs and correspond to colors used in Figures 2 and 3. Grey circles refer to ant mounds where a focal species was not sampled in a particular year. Data are presented for those mounds in which the same species was found in at least two of the three consecutive years.

Ten of the ant mounds sampled in 2008 were resampled in 2009 and 2010. In seven of these we found one or more of the focal species in the subsequent years (Figure 5). Most MLGs that we found in later years had already been found in the same mounds in 2008. There were only two exceptions to this apparent continuity over time: in the first mound resampled for *T. ulmi* (Figure 5b) we found an additional MLG in 2009 that had not been observed in that mound in the previous year, and in the second mound resampled for *F. marginata* (Figure 5c), we found a MLG that had not been identified before, but which belonged to one of the MLLs that had been observed in 2008 in other nest mounds nearby (colored in green shade, Figure 2). These apparent exceptions might either reflect recent colonization events or might be due to under-sampling in 2008. For example, the overall composition of the nest mound in which the *F. marginata* MLG was newly observed did not change significantly between 2008 and 2009 (Fisher Exact Test, P = 0.111), likely because the newly observed MLG belonged to a MLL that had a population-wide frequency of 0.044 in 2008. With such a low frequency, it is quite likely that this MLG was missed in an earlier year. In contrast, the overall composition of aphid MLGs in the nest where we found a new MLG for *T. ulmi* did significantly change between 2008 and 2009 (Fisher Exact Test, P = 0.024). It thus appears less likely that the new MLG was due to under-sampling in 2008, since this MLG occurred at a high frequency overall (0.52). Overall, we infer that clonal lineage composition of aphid livestock in *L. flavus* ant mounds changes relatively little from year to year. We would have liked to test this with a formal heterogeneity analysis across years, but too low numbers in several cells precluded this.

## Discussion

### Aphid distribution and abundance

In half of the ant mounds sampled in 2008 only one of the three focal root aphid species, *G. utricularia, T. ulmi* or *F. marginata* was found, despite the other aphid species being present within a radius of 50 m. This level of aphid specificity among ant nests matches earlier findings by [31] in a British field survey of the same ant species and its underground aphids. If there were multiple aphid species per mound, we found that they tended to be clustered in separate soil samples and hardly ever occurred in the same aphid chamber. This not only applied for the three most abundant species that we focused on, but also for other rarer species of root aphids. We are confident, therefore, that inclusion of these other aphids would not have changed our overall conclusions. Unfortunately, aphid sample sizes remained low for most mounds and for two of the three focal species, which seems unavoidable as earlier non-destructive large-scale surveys obtained similar numbers of adult root aphids for these species ( Additional file 1). Comparing frequencies and absolute densities across studies is not easy as authors have used different sampling methods in the past [33,37]. While these have given very different estimates of root aphid density, we show in the Additional file 1 that this is almost certainly due to these sampling differences, and that our estimates of adult aphid densities are in line with previous studies. In spite of these sample size limitations, we are confident, therefore, that our results would be repeatable with larger sample sizes at: (1) the mound level, because aphids were generally found scattered throughout the entire mound, so that systematic bias in our non-destructive sampling appears unlikely, and (2) at the chamber level, because Figure 4 shows that within-chamber aphid diversity does not increase with increasing numbers of aphids per chamber (ca. equivalent to chamber size). Moreover, in our statistical analysis we control for any effect of the low sample sizes, by simulating the exact same sample sizes as achieved in the field.

The considerable interaction-specificity, often between single ant colonies and single aphid lineages was also encountered at the genetic level within species. Mounds often only harbored one clonal lineage of a single aphid species and if mounds had multiple aphid clones they were almost always compartmentalized in different chambers. A similar degree of host specificity has also been shown for above-ground aphids tended by ants as opposed to non-tended aphids [38]. However, complete spatial separation of aphid clones is less frequently observed above ground [38,39], probably because these aphids can more easily move around. Our limited sampling across years further indicated a high degree of constancy of distributions of aphid clones over time. It would have been interesting to compare our results with similar studies on other myrmecophilous and non-myrmecophilous root aphids, but to our knowledge such studies have not yet been done.

### Within- and between ant mound aphid distribution patterns

The between- and within-mound distribution patterns were very similar for the three aphid species under study (Figure 2), suggesting that similar dispersal and recruitment mechanisms apply. Most aphid chambers contained only a single aphid (Figure 4) and chamber sizes seemed proportional to the number of aphids housed in them ( Additional file 1). It thus appears unlikely that these aphids competed for limiting phloem resources, even in the few cases where different aphid species or MLLs shared a chamber. Rather, the husbanding ants seem to optimize the feeding conditions for each aphid adult, because aphid densities (on average 1.00 per liter soil, Additional file 1) remained well below densities that would occupy all available root phloem resources. These relatively low numbers of adult aphids can be explained by the ants eating the vast majority of aphid nymphs and only keeping a small number of adults for honeydew production as inferred previously by [33].

The low aphid diversity per mound, the apparent invariance of clonal distributions per mound among years, and the high degree of population viscosity [14] are consistent with horizontal transmission of aphids between mounds being infrequent. After successful dispersal and adoption, aphid propagation within mounds would then mostly be in the form of clonal copies of fundatrices (aphid ‘foundresses’) replacing their ancestors. We would thus expect that the genetic diversity of aphid livestock within a given ant mound would slowly increase over the years. The densest *L. flavus* populations in Northwest Europe are normally found in extensively grazed old pastures that have been stable for centuries and where nest-mounds are large because many generations of *L. flavus* colonies have contributed to building them. Compared to such populations, the coastal transect that we studied is more variable in age and stability, which appeared to be reflected in the younger parts of the salt-marsh harboring less aphid diversity, at least for *G. utricularia* ( Additional file 1). Patterns like this would be reminiscent of older trees having richer communities of underground mycorrhiza and leaf-endophytes [40,41], but also of above-ground aphid colonies becoming more genetically diverse over the season due to the immigration of new aphid clones [39].

### Inferring the evolutionary logic of aphid husbandry in *Lasius flavus* colonies

Genetic diversity of symbionts has been a central issue in mutualism theory [3,21,22] as diversity levels that simultaneously maximize the fitness of both hosts and symbionts are often expected to be low [21,22]. This is indeed what we found throughout our data set (i.e. at the species, MLL and MLG level). Compartmentalization of symbionts is known to promote mutualism stability in other systems (e.g. mycorrhizal mutualisms [42]), because benefits can be preferentially allocated toward cooperative symbionts. However, many of these conceptual arguments are based on the assumption that symbiont lineages compete and that the collateral damage of such interactions for host fitness maintains selection to suppress symbiont diversity [22]. While the high root aphid densities per *L. flavus* mound reported in the literature ( Additional file 1) that inspired this study suggested that such competition might also apply in this system, our results prompt us to reappraise this assumption, because: 1. Aphid husbandry is special, relative to other resource enhancing mutualisms, in that the ant hosts can exploit their aphid symbionts both for sugars (“milking” adults in their prime age) and for proteins (eating young instars and old adults) and 2. Our data suggest that consumption of most of the aphid offspring by the ants reduces total aphid numbers per mound ( Additional file 1) to such extent that the grass-root phloem resource constraints that might have induced aphid competition are unlikely to apply.

Many details of the interaction between *L. flavus* ants and their communities of mutualistic root aphids remain unknown and deserve further study. However, our present results indicate that the biological details and specific resource constraints of an obligate mutualism may be decisive for the selection factors that determine evolutionary stability over time. Our present data indicate that prevailing paradigms of partner choice and sanctions [5,10,24,43] may not apply in the ant-aphid mutualism that we studied, because fundamental assumptions of scramble competition between unrelated symbionts [22] are not fulfilled ( Additional file 1).

After initial domestication, the aphid clones would have continued to benefit from the symbiosis, because the premature death of most early instar nymphs (which individually are of low value as sugar providers for the ants) reduces competition over resources and reproduction, and extensive clonality ensures that vertical transmission will maintain clonal tenure within nests. This interpretation might explain why *L. flavus* is reputedly obligately dependent on root aphids [33,35], but without having specialized on any of the large number of aphid species that are available, despite the aphids having evolved specialized traits that enhance productivity as ant symbionts but preclude independent life (see [14] for details). Testing the validity of our interpretation that early instar aphids are worth more as direct sources of protein than as later sources of carbohydrates will require controlled lab experiments, which might be feasible in spite of the challenges of keeping these ants and aphids in artificial nests [44].

### Analogies with human subsistence farming

The results of our study suggest that polyculture aphid husbandry in *L. flavus* follows similar efficiency principles as modern cattle husbandry practices in humans, where adult cows are kept in numbers that secure maximal milk-productivity in a competition-free environment and where surplus reproduction is slaughtered for meat-consumption soon after birth. How this analogy could come about is interesting to evaluate.

The English name for *L. flavus*, Yellow meadow ant, indicates lack of pigmentation because the ants are almost never exposed to direct sunlight. This exclusively underground life style, shared by many but far from all *Lasius* ants [35], must have implied that foraging territories became limited to the direct nest environment, so that access to prey was reduced but protection and monopolization of domesticated aphids became easier. Intensification of aphid husbandry thus seems a logical consequence of becoming subterranean and a prudent way of harvesting a small local resource-base that ultimately depends on primary production (grass roots) rather than secondary production (free-living prey capture). Extensive culling of immature aphids for meat not only allowed polyculture practices (by eliminating competition), but may have actively encouraged it when different aphid species would exploit somewhat different plant root niches, when their availability would be unpredictable, or when they would produce honeydew with slightly different chemical composition [45].

The analogies between aphid husbandry in *L. flavus* and human cultural practices are quite striking as farming husbandry allowed human populations to sustain themselves at much higher densities than hunter-gatherer populations [46]. Likewise, the density of *L. flavus* ants in mature grasslands is among the highest known for ants [35,47,48] and appears to be sustainable with only a modest ecological footprint. As in humans, the secret of success appears to be a unique combination of traits, such as the ability to actively engineer nest mound habitat (a form of niche construction [49]) rather than living in fixed plant structures as other obligately aphid-dependent ants do (e.g. [20]), and the availability of multiple aphids that could be domesticated without the need to specialize on any one of them. This suggests that ant farming practices for meat [50,51] deserve more explicit study, as they may provide remarkable insights into sustainable farming practice.

## Conclusions

Farming mutualisms are highly diverse. Some have a long history of coadaptation, specificity and vertical symbiont transmission, whereas others have evolved interdependences based on horizontal symbiont acquisition and low specificity. Many ant species obtain facultative benefits from tending aphids. Some of these interactions have evolved to be highly specific, but the *Lasius flavus* husbandry system that we studied is unusual in that both ants and root aphids appear to be obligately interdependent and adapted to their respective life styles as farmers and livestock, but without obvious signs of species-by-species interaction specificity.

Our genetic explorations of a large island population with dense populations of *L. flavus* suggest that the combination of permanently underground nesting, aphid clonality, and very low gene flow between aphid populations of neighboring mounds has allowed these ants to evolve an unusual form of polyculture symbiosis. Species and clonal lineages of aphids appear to be kept apart, which likely gives colonies the possibility to actively manage the diversity and abundance of their livestock. We hypothesize that this allows the ants to secure maximal yield from a subset of mature aphids that are kept for carbohydrates under optimal conditions of phloem feeding and ant care. These selected aphids may then also reproduce at the highest possible rate, so that the ants both secure maximal protein intake by eating the excess of early instar aphids, and replacement of their honeydew-producing livestock when adult aphids age and become less productive.

Many mechanistic details that govern the dynamics of this mutualism await further research. However, we feel that analogies with human husbandry practices based on similar cost-benefit considerations lend sufficient credibility to our interpretations to generate novel interest into natural selection processes that have produced ant farming practices for both meat and carbohydrates.

## Methods

### Natural history of the model system

The subterranean Yellow meadow ant *Lasius flavus* constructs conspicuous nest mounds (↕ ca. 30 cm, ø ca. 80 cm) in grassland habitats to house both its own colonies and the root aphids on which it depends for honeydew as a source of carbohydrates [18,30,32,33] and which they eat for protein [30,32,33]. The ants actively protect the aphids ( [52], A.B.F. Ivens, personal observation) and keep them in specially constructed ‘aphid-chambers’, cavities around grass-roots with one or several aphids (Figure 1a). Thirteen species of root aphids are known to be tended by *L. flavus*, often with multiple species in the same nest mound ( [18,31,33,34], A.B.F. Ivens, personal observation). Among these, *Tetraneura ulmi, Geoica utricularia* and *Forda marginata* are often the most dominant species [14,18,31,33,34]. This was also the case at our study site, so we focused our study on these three species. These aphids can also be found in nests of other ants, such as *Myrmica sp.* and other *Lasius* species [18], albeit in lower numbers than in the typical *L. flavus* mounds.

Aphid reproductive cycles can be fully asexual (anholocyclic) or include a single sexual phase at the end of the season (holocyclic). In another study [14] we showed that the three focal aphid species are predominantly if not completely asexual in our study population (see also below), consistent with all three species having been shown to feed year-round on roots of the grasses *Festuca rubra, Agrostis* spp. and *Elytrigia maritima* without requiring a host shift during winter [14,18,31,33]. The possible winter host shift to *Ulmus* trees that has previously been described for *Tetraneura ulmi* ( [18], O.E. Heie, personal communication) thus appears to be absent in our NW European study population. However, several other mechanisms can account for more limited horizontal aphid dispersal in salt march habitats such as our study site: walking, floating on tidal floods and wind dispersal of winged individuals (alates) that are produced at very low frequencies in all three species. Considerable genetic population viscosity confirms that horizontal dispersal between mounds is generally limited [14]. However, this appears to be the only dispersal mode available as neither vertical nor horizontal transmission by the tending workers has ever been observed for *Lasius* ants.

### Sampling methods

Ant mounds were sampled for aphids on the island of Schiermonnikoog, the Netherlands (53°28′ N, 6°09′ E) in July 2008, 2009 and 2010 along a 7 km transect across most of the salt-marsh on the island (Figure 1b). The westernmost first kilometer close to the inhabited part of the island was grazed by cattle, whereas the remaining transect crossed ungrazed salt-marsh. The transect was subdivided into eight locations (one every km). At every location we sampled five same-sized ant mounds (Ø ca. 60 cm), taking 21 cylindrical soil samples (10 cm deep and Ø 8 cm, volume 0.64 l), according to a fixed sampling scheme (Figure 1b). The average volume of the part of the mounds that was suitable for aphids (i.e. had roots of the appropriate grasses) was 66.7 l ( Additional file 1). We obtained this estimate by adding the volume of the aboveground part of an average mound and the volume of a ring directly surrounding the mound (10 cm wide, 8 cm deep) which is known to often contain root aphids as well [33,34].

Every soil sample was hand-sorted for ‘aphid chambers’, cavities containing one or more root aphid individuals in spatial isolation from any other aphids (Figure 1a). This sampling scheme resulted in a four-level nested design: transect location, ant nest mound, soil sample and aphid chamber (Figure 1b). In July 2009 and 2010 we resampled 10 of the 40 previously sampled nest mounds, for which we had obtained sufficiently detailed aphid distributions in 2008 to be able to detect changes in later years.

### Molecular methods and data analysis

A detailed description of the molecular analysis of the aphids and properties of the genetic markers is provided by [36]. In short, all collected aphids were genotyped for an array of polymorphic microsatellite markers (*Geoica utricularia,* eight markers: Gu2, Gu3, Gu5, Gu6, Gu8, Gu9, Gu11, Gu13; *Forda marginata*, seven markers: Fm1, Fm3, Fm4, Fm6, Gu6, Gu11, Gu13; *Tetraneura ulmi*, six markers: Tu1, Tu2, Tu3, Tu4, Tu10, Tu11) after DNA extraction from entire aphids using 200 μl 2yChelex® 100 resin (Fluka) [53]. Following PCR-amplification, products were analyzed on an ABI-PRISM 3130XL (Applied Biosystems) sequencer and chromatograms were analyzed in Genemapper (Applied Biosystems).

When amplification failed, samples were re-run at least two more times. When amplification remained unsatisfactory, the specific microsatellite locus was scored as ‘missing data’. When data were missing for more than half of the loci the individual was omitted from further analysis. In total, we included 239 individuals of *Geoica utricularia* (2008: 201, 2009: 23, 2010: 15, after omitting a total of 28 individuals), 191 of *Forda marginata* (2008: 158, 2009: 4, 2010: 29, 11 omitted) and 105 of *Tetraneura ulmi* (2008: 92, 2009: 7, 2010: 6, 4 omitted).

Diploid clonal multilocus genotypes (MLGs) consist of a unique combination of alleles across all genotyped loci. The genotypic data allowed us to assign every aphid to a MLG using the software MLGSIM2.0 [54], an updated version of MLGSIM [55]. A multilocus lineage (MLL) is a group of closely related MLGs that differ by only one or two alleles [14]. All MLGs could be grouped into MLLs. The complete analysis is detailed in [14].

When a sample only contained aphids from a single species, MLL or MLG, we classified that sample as a ‘monoculture’ at the species, MLL or MLG level. Samples were taken at three ‘sampling levels’: ant mound, soil sample or aphid chamber. To test whether the observed monocultures occurred more frequently than expected under a random distribution, we wrote a bootstrap routine in R 2.13.0 [56] (routine available upon request). For a given level of sampling, the routine distributed the species, MLLs, or MLGs randomly over samples in 1000 iterations with simulation sample sizes corresponding to the observed sample sizes. The routine thus used the observed frequency distributions of species, MLLs, or MLGs at the sampling level above the focal level (Figure 1b) to estimate the probability (P) that the same or a higher number of monocultures than the observed number would be obtained by chance (one-tailed test). When P was < 0.05, the null hypothesis that the observed number of monocultures resulted from a random distribution of aphids over samples was rejected.

Any behavioral experiments referred to were conducted with insects. In this context, insects are not considered animals, so these experiments do not, as far as we are aware, require an formal approval of an ethics committee.

## Competing interests

The authors declare that they have no competing interests.

## Authors’ contributions

ABFI and JJB designed research with input from IP and FJW. ABFI did the fieldwork and the genetic analyses, the latter with contributions from DJCK, both for the practical work and the final analyses. ABFI conducted the statistical analyses, with contributions from IP. ABFI and JJB wrote the paper, with several rounds of contributions from DJCK, IP and FJW. All authors read and approved the final manuscript.

## Authors’ information

All authors have long standing interest in the evolutionary biology of cooperation and conflict within and between species. JJB, DJCK and ABFI mainly focus on different aspects of social evolution of ants and their mutualistic partners. FJW and IP are theoretical biologists, focusing on the theory of conflict and cooperation in social systems.

## Supplementary Material

Additional file 1 **This file provides additional information on (1) Aphid abundance estimates (including a table with present and previous estimates) (2) Absence of scramble competition in the study system and (3) Ecological factors influencing aphid diversity**[57-60]. Click here for file

## Author details

^1^Theoretical Biology, Centre for Ecological and Evolutionary Studies, University of Groningen, Nijenborgh 7, Groningen 9747 AG, The Netherlands.^2^Centre for Social Evolution, Department of Biology, University of Copenhagen, Universitetsparken 15, Copenhagen DK-2100, Denmark.^3^Laboratory of Insect Social Evolution, The Rockefeller University, 1230 York Avenue, New York, NY 10065, USA.
