# Supplementary material for: Ants farm subterranean aphids mostly in single clone groups - an example of prudent husbandry for carbohydrates and proteins?
Source: BMC Evol Biol. 2012 Jul 2;12:106. doi: 10.1186/1471-2148-12-106 (PMC3499235; doi:10.1186/1471-2148-12-106)
Supplement: Additional file 1 — This file provides additional information on (1) Aphid abundance estimates (including a table with present and previous estimates) (2) Absence of scramble competition in the study system and (3) Ecological factors influencing aphid diversity[57-60]. [file 1471-2148-12-106-S1.pdf]

Additional File

Aphid abundance estimates

The aphid numbers in our samples were low relative to the numbers that have previously been estimated for *L. flavus* territories (5500-17000 aphids per ant nest of medium size, i.e. 23000 ants) [33, 37]. However, neither in previous surveys nor in the present study were mounds sampled exhaustively, in order to preserve them for later resampling. This implied that aphid density estimates could only be based on extrapolations from aphid numbers in soil core samples, which in our study covered on average 20% (range 11.4%-62.8%) of the estimated total volume of ant mounds that was suitable for aphid-culture.

We collected 14.5 ± 2.07 (mean ± s.e.) adult aphids of the focal species per nest, which produced an estimate of the cumulative total adult aphid population per mound for the three focal root aphid species of 67 individuals (range 18-134) (Table S1). Most of the discrepancies with earlier estimates appear to be due to previous studies using Tullgren funnel extraction methods, so that all developmental stages were collected over a period of several days, whereas we used hand-sorting that only allowed collection of adult aphids and occasionally fourth instar nymphs (Table S1). The differences in numbers obtained will likely have been further enlarged by the fact that aphids will continue to give birth during the 4-5 day long Tullgren extraction, with many of them ending up in the collection vials instead of being eaten by the ants [33]. Moreover, earlier authors included a larger part of the potential ant territory and sampled ten root aphid species more than we were able to analyse genetically. Approximate corrections for these possible sampling biases produced density figures of adult aphids per litre of mound-soil that were much closer to our present findings (Table S1).

On average 88% of the root aphids that previous authors collected by Tullgren funnel extraction belonged to the nymphal stages that our hand sampling method missed. It therefore seems reasonable to assume that almost all of these never become established in chambers as carbohydrate providers to the ant society, but were eaten by the ants before they become adult [33]. This would imply that population numbers of adult root aphids remain well below the density levels that would exploit all available phloem resources that could possibly be accessed via grass roots. Whether *L. flavus* indeed balances its preying behavior based on carbohydrate intake would need further testing in controlled laboratory experiments [33] [44]. A result consistent with this hypothesis would seem likely, because a shift from milking to preying behavior has been documented for *L. niger* after workers were offered a carbohydrate food supplement [50]. *L. niger* belongs to the same genus as *L. flavus* and often lives in the same grasslands habitats where it avoids competition with *L. flavus* by foraging above ground (Pontin [48, 57, 58]).

Further arguments for the likely absence of scramble competition between root aphids

Of the total of 239 opened aphid chambers that were inhabited by the three focal species in 2008, only 92 (38.5%) contained more than one aphid (range 2-13). Within this subsample only a single chamber contained aphids of two species and only 11 chambers (4.6%) contained 2 MLLs (Figure 4). Aphid chambers are small cavities that are excavated by the ants alongside roots of grasses like *Festuca rubra* and *Elytrigia maritima.* Although chamber volumes were not measured, they often seemed approximately proportional to the number of aphids housed in them, suggesting that the ants expand chambers when they need to contain more adult aphids and more roots for these aphids to extract phloem sap from. Combined

with the abundant availability of grass roots in *L. flavus* mounds and our average yield of ca. 1 adult aphid per litre soil (Table S1), this minimal coexistence with non-clone mates and the absence of chamber space constraints would make it very unlikely that individual ant-tended aphids would not have access to ad libitum phloem resources.

External factors that may affect aphid diversity at a larger scale

Overall, we would expect that the genetic diversity of aphid livestock would tend to slowly increase when *L. flavus* mounds become larger over the years of their existence, but we did not have a range of mound-size data to test this and neither are we aware of directly relevant data on this by others. However, the transect locations that are known to be the oldest from historical records about salt marsh development on the island of Schiermonnikoog (locations 1,2 and possibly 7) [59], harbored mounds that yielded a higher aphid diversity, at least for *G. utricularia* for which we had most data. Similarly, mounds occurring at lower elevations will be more frequently subjected to flooding, a disturbance that might cause mounds to be growing slower and have longer periods without abundant ant habitation [60]. Also this seems at least partially consistent with our data, as aphid clonal diversity in mounds on transect locations with lower elevation levels (e.g. location 5 and 6; Ivens et al, unpublished data), tended to have lower clone diversity with the exception of *F. marginata*). Aphid numbers of each of the three species varied considerably across the transect, possibly reflecting subtle differences in local ecological conditions related flooding frequency and salinity owing to slight elevation differences (Ivens et al., unpublished data). None of these differences appear to have affected the overall results and conclusions of our study, but they may be of interest for future ecological studies of *L. flavus* populations in coastal areas.

Table S1 Observed and estimated number of aphids per litre of soil in sampled mounds of *L. flavus* in different studies

Study	Observed number of aphids/liter soil (summer)	% focal species	% adults	Corrected number of aphids/liter soil			Estimated mound volume (l)		Original estimated total number of aphids/mound	Corrected estimated total number of aphids/mound	
				Mean	Range		Mean	Range		Mean	Range
Pontin 1978	32.9[1]	53.3	8.4	1.48	-		85.00	-	12700	125.80	-
Godske 1992	48.3	28.7	15.0	2.08	-		56.00	-	5506	116.48	-
Present study	1.73[2]	63.9[2]	92.1[2]	1.00	0.86 - 1.14		66.76	21-117	-	66.99	18.06 – 134.12

[1]Observed numbers are given per soil sample [33]. We estimated the volume of these soil samples to be 1.13 l and corrected observed numbers accordingly

[2]Based on adults and fourth instar nymphs only

Observed numbers of aphids per liter of soil inferred from the available literature are given only for July and August, as that period corresponds to the sampling scheme applied in the present study. We estimated percentages of observed adults of *G. utricularia, T. ulmi* and *F. marginata* after correcting original total numbers [33, 35, 37] by the cumulative percentage of these three species relative to all root aphids (older studies found 4-5 more species, which we ignored) and by adjusting for the percentage of adults (close to 100% in our study and much less in the other studies). This produced the final estimates of the number of adult aphids of these three species per liter of soil and per mound, showing that numbers are roughly comparable.
